# The Effects of Caffeine Supplementation on Physiological Responses to Submaximal Exercise in Endurance-Trained Men

**DOI:** 10.1371/journal.pone.0161375

**Published:** 2016-08-17

**Authors:** Mark Glaister, Benjamin Henley Williams, Daniel Muniz-Pumares, Carlos Balsalobre-Fernández, Paul Foley

**Affiliations:** 1 School of Sport, Health, and Applied Sciences, St Mary’s University, Strawberry Hill, Twickenham, United Kingdom; 2 Department of Physical Education, Sport & Human Movement, Autonomous University of Madrid, Madrid, Spain; 3 Cardiff School of Health Sciences, Cardiff Metropolitan University, Cardiff, United Kingdom; University of Rome, ITALY

## Abstract

**Objectives:**

The aim of this study was to evaluate the effects of caffeine on physiological responses to submaximal exercise, with a focus on blood lactate concentration ([BLa]).

**Methods:**

Using a randomised, single-blind, crossover design; 16 endurance-trained, male cyclists (age: 38 ± 8 years; height: 1.80 ± 0.05 m; body mass: 76.6 ± 7.8 kg; $\dot{V}{\text{O}}_{2\max}$: 4.3 ± 0.6 L∙min^-1^) completed four trials on an electromagnetically-braked cycle ergometer. Each trial consisted of a six-stage incremental test (3 minute stages) followed by 30 minutes of passive recovery. One hour before trials 2–4, participants ingested a capsule containing 5 mg∙kg^-1^ of either caffeine or placebo (maltodextrin). Trials 2 and 3 were designed to evaluate the effects of caffeine on various physiological responses during exercise and recovery. In contrast, Trial 4 was designed to evaluate the effects of caffeine on [BLa] during passive recovery from an end-exercise concentration of 4 mmol∙L^-1^.

**Results:**

Relative to placebo, caffeine increased [BLa] during exercise, independent of exercise intensity (mean difference: 0.33 ± 0.41 mmol∙L^-1^; 95% likely range: 0.11 to 0.55 mmol∙L^-1^), but did not affect the time-course of [BLa] during recovery (*p* = 0.604). Caffeine reduced ratings of perceived exertion (mean difference: 0.5 ± 0.7; 95% likely range: 0.1 to 0.9) and heart rate (mean difference: 3.6 ± 4.2 b∙min^-1^; 95% likely range: 1.3 to 5.8 b∙min^-1^) during exercise, with the effect on the latter dissipating as exercise intensity increased. Supplement × exercise intensity interactions were observed for respiratory exchange ratio (*p* = 0.004) and minute ventilation (*p* = 0.034).

**Conclusions:**

The results of the present study illustrate the clear, though often subtle, effects of caffeine on physiological responses to submaximal exercise. Researchers should be aware of these responses, particularly when evaluating the physiological effects of various experimental interventions.

## Introduction

Caffeine, a trimethylxanthine, is one of the most commonly consumed drugs in the world, with no apparent long-term adverse health effects [1]. Research into the ergogenic properties of caffeine has revealed benefits across a range of exercise intensities and durations, with the greatest effects being displayed in sustained high-intensity aerobic activities [2,3]. Indeed, typical doses of 3–6 mg∙kg^-1^ ingested 30–90 minutes prior to exercise have produced positive effects (0.7–5.4%) in time trial events lasting 5–60 minutes [4–15]. Nevertheless, the mechanisms to explain the beneficial effects of caffeine supplementation on exercise performance remain unresolved. Although early research supported a glycogen-sparing mechanism of action, the absence of a corroborative change in respiratory exchange ratio (RER), combined with evidence of significant effects in relatively short events (< 60 minutes) where glycogen availability would not be a limiting factor [3], has led researchers to consider alternative explanations. At present, although the lipophilic properties of caffeine leave room for a possible small direct intracellular effect on muscle function [16], the key mechanism by which caffeine is believed to enhance sustained high-intensity performance is via a central mechanism involving the antagonism of adenosine receptors and leading to increases in neurotransmitter release, motor unit firing rates, and pain suppression [17].

Most studies have attempted to explain the beneficial effects of caffeine supplementation on sustained high-intensity exercise by evaluating corresponding changes in various physiological responses such as heart rate, blood lactate [BLa], oxygen uptake ($\dot{V}{\text{O}}_{2}$), RER, etc. However, since most of those studies have observed a caffeine-induced improvement in exercise performance, it is difficult to distinguish the direct effects of caffeine on physiological responses from those associated with the improved performance. Although several studies have attempted to address this problem by including a fixed-intensity submaximal bout of exercise (generally at around 60–80% $\dot{V}{\text{O}}_{2\max}$) prior to a performance-based test, and often as part of a warm-up, the results contain some discrepancies. For example, whilst some studies have found no effect of caffeine on minute ventilation (${\dot{V}}_{\text{E}}$) [18–20], others have reported a significant increase [4,7]. Similarly, many studies report no effect of caffeine on $\dot{V}{\text{O}}_{2}$ [4,5,7,9,13,14,18,20–23], though some have reported a significant increase [5,15].

One particularly strange effect that often accompanies caffeine supplementation, and thereby providing further evidence against a glycogen-sparing mechanism of action, is an elevation in [BLa] during exercise (3). The effect is strange because, although the increase in [BLa] can often be explained by a concomitant caffeine-induced increase in performance [5–8,11, 13,14,22], this is not always the case [24]. Moreover, while some studies have found no effect of caffeine supplementation on [BLa] during submaximal exercise [4,13–15,18–20,25,26], others have observed a significant increase [5,9,21,22]. To complicate matters further, Graham et al. [21] could not attribute the caffeine-induced increase in [BLa] during submaximal exercise to an increase in production or release by the active muscles. In effect, this leaves four possibilities; first, that the methods used by Graham et al. [21], though more complex than most, lacked sufficient sensitivity to detect changes in muscle lactate production and release resulting from caffeine supplementation; secondly, that lactate is being produced from inactive muscle; thirdly, that caffeine impairs [BLa] clearance; and finally, that those studies finding a caffeine-induced increase in [BLa] were, in some way, affected by methodological limitations (i.e. limited sample size, or a failure to control cadence). Of those possibilities, the idea that the caffeine-induced increase in [BLa] could be due to an impairment of lactate clearance is plausible, given that gluconeogenesis accounts for 20–30% of lactate clearance during exercise [27], and that adenosine signalling has been shown to stimulate gluconeogenesis, at least in animal models [28]. Nevertheless, the ubiquitous nature of adenosine receptors coupled with their ability to activate and inhibit the same signalling cascades [28] makes it difficult to identify the precise mechanism by which caffeine influences [BLa]. The aims of the present study, therefore, were to evaluate the effects of caffeine supplementation on physiological responses to submaximal exercise, with a particular focus on the effects on [BLa]. It was hypothesised that caffeine would increase [BLa] during exercise and slow its rate of clearance during recovery.

## Materials and Methods

### Participants

Sixteen endurance-trained [29], competitive, male athletes (cyclists and triathletes) volunteered for the study which was approved by St Mary’s University Ethics Committee. Prior to testing, participants received written and verbal instructions regarding the nature of the investigation and completed a training history questionnaire, which indicated that all had been actively involved in sport for approximately 19 ± 10 years and that, at the time of the investigation, the distance cycled each week was 211 ± 65 km. Prior to commencement, all participants completed a health-screening questionnaire and provided written informed consent. Means ± standard deviation for age, height, body mass, body fat, and $\dot{V}{\text{O}}_{2\max}$ of the participants were: 38 ± 8 years, 1.80 ± 0.05 m, 76.6 ± 7.8 kg; 17.5 ± 3.5%, and 4.3 ± 0.6 L∙min^-1^ (55.6 ± 5.0 ml∙kg^-1^∙min^-1^), respectively. Participants were instructed to maintain their normal diet throughout the testing period, to follow the same diet for 24 hours prior to each trial, to avoid food and drink in the hour before each trial, and to refrain from strenuous exercise for 24 hours before each trial. Participants were provided with a list of dietary sources of caffeine and asked to refrain from consuming these for 24 hours prior to each trial. A questionnaire was used to establish normal daily caffeine intake.

### Procedures

All trials were completed at approximately the same time of day in a laboratory which was thermostatically controlled at 19°C. On arrival at the laboratory, and after approximately five minutes of seated rest, a resting blood sample (~ 5 ml) was drawn from a branch of the basilic vein and collected in a lithium-heparin tube (Vacutainer; Becton Dickinson, Oxford, United Kingdom). At the same time, a blood sample was obtained from the earlobe via capillary puncture for the evaluation of [BLa] via an automated analyser (Biosen C-Line; EKF Diagnostic, Ebendorfer Chaussee, Barleben, Germany). In Trial 1, participants subsequently had their body fat content evaluated using a four-site skinfold protocol [30], before completing a submaximal incremental step test on an electromagnetically-braked cycle ergometer (Lode Excalibur Sport; Groningen, Holland). The cycle ergometer was fitted with clipless pedals and participants cycled using their own cycling shoes. Prior to Trial 1, the ergometer was adjusted (saddle height and handlebar position) for each participant and the settings were noted for replication in trials 2–4. The starting intensity and increment size for each participant for Trial 1 was estimated, based upon feedback from participants regarding their typical race pace, to achieve a [BLa] profile consisting of 5–8 stages beginning at a [BLa] of around 1 mmol∙L^-1^ and ending with a [BLa] > 4 mmol∙L^-1^. The duration of each increment was 3 minutes, and a 30 s break was provided at the end of each stage to enable [BLa] to be evaluated. To limit any effect of cadence on the [BLa] response to exercise [31], participants were given 30 s during the first stage of Trial 1 to achieve a comfortable cadence and were instructed to maintain this throughout all trials. Heart rate was monitored at 5 s intervals throughout all trials using a heart rate monitor (Polar s610i; Polar Electro Oy, Kempele, Finland), and ratings of perceived exertion (RPE) were recorded 30 s from the end of each incremental stage using a 15-point scale [32]. After five minutes of passive rest, participants completed a second incremental test (Trial 1 only), using the same starting intensity and increment size; however, for this phase of the trial the duration of each increment was reduced to 1 minute. The test was terminated when participants reached volitional exhaustion, at which time a final [BLa] measurement was obtained. Oxygen uptake was monitored (breath-by-breath) throughout all trials using an on-line gas analyser (Oxycon Pro; Jaeger, Hoechberg, Germany). The analyser was calibrated before each trial using oxygen and carbon dioxide gases of known concentrations (Cryoservice; Worcester, UK) and the flowmeter was calibrated using a 3-litre syringe (Viasys Healthcare GmbH; Hoechberg, Germany). During all trials participants breathed room air through a facemask (Hans Rudolph; Kansas City, MO, USA) that was secured in place by a head-cap assembly (Hans Rudolph; Kansas City, MO, USA). $\dot{V}{\text{O}}_{2\max}$ was determined as the highest 30 s average $\dot{V}{\text{O}}_{2}$ recorded during the second incremental test of Trial 1 provided that at least two of the following criteria had been met: 1) A plateau in $\dot{V}{\text{O}}_{2}$; as determined by an increase of less than 2 ml∙kg^-1^∙min^-1^ over the previous stage; 2) A RER ≥ 1.15; 3) A heart rate within 10 b∙min^-1^ of age predicted maximum; 4) A [BLa] ≥ 8 mmol∙L^-1^.

In trials 2–4, after the first venous blood sample was obtained, participants ingested a gelatine capsule containing either 5 mg∙kg^-1^ of caffeine (My Protein; Manchester, UK), or placebo (maltodextrin: My Protein; Manchester, UK). After supplementation, participants rested for 40 minutes before the same blood sampling procedure was repeated. In trials 2 and 3, using a single-blind balanced randomised crossover design, participants completed subsequently a six-stage submaximal incremental test with Stage 3 set at the power output required to elicit the gas exchange threshold (GET) and Stage 6 set at the power output required to elicit the onset of [Bla] accumulation (OBLA); the latter equating to a [Bla] of 4 mmol∙L^-1^ [33]. The GET was estimated from visual inspection of the $\dot{V}{\text{CO}}_{2}-\dot{V}{\text{O}}_{2}$ relationship, established in Trial 1, using the V−slope method [34]. OBLA was identified using software (Lactate-E) specifically developed for the purpose [35]. Prior to start of each incremental test, participants sat passively on the cycle ergometer for 3 minutes to enable resting measures (determined from the final 30 s) of $\dot{V}{\text{O}}_{2}$, RER, ${\dot{V}}_{\text{E}}$, breathing frequency (BF), and heart rate to be recorded. Upon completion of each incremental test, participants rested passively for 30 minutes. As in Trial 1, [BLa] was evaluated at the end of every incremental stage, but also at 2 minute intervals during recovery. $\dot{V}{\text{O}}_{2}$, RER, ${\dot{V}}_{\text{E}}$, BF, heart rate, and RPE were monitored throughout all incremental tests and during recovery. Respiratory data were filtered by removing values (attributed to noise) that were outside three standard deviations of the local mean, linearly interpolated to provide values at 1 s intervals, and averaged over the final 30 s of each incremental stage to provide a mean response for each exercise intensity. Respiratory data were also averaged over the final 30 s of each post-exercise recovery period to determine whether any effects of caffeine at rest returned during recovery.

The step incremental protocol for Trial 4 was determined by evaluating which of trials 2 and 3 produced an end-exercise [BLa] closest to 4 mmol∙L^-1^. If the answer was the placebo trial, then caffeine was administered as the supplement in Trial 4, and the results of the previous caffeine supplemented trial were used to adjust the protocol (again evaluated using the Lactate-E software) to achieve an end-exercise [BLa] close to 4 mmol∙L^-1^. If, on the other hand, caffeine produced the end-exercise [BLa] closest to 4 mmol∙L^-1^ then Trial 4 became a placebo supplemented trial, with the protocol adjusted accordingly based on the results of the previous placebo trial. In effect, Trial 4 was designed to evaluate the effects of caffeine on [BLa] clearance during passive recovery from a fixed starting [BLa] of approximately 4 mmol∙L^-1^. The recovery kinetics of [BLa] were evaluated by fitting mono-exponential models (Eq 1) to the data, using a non-linear least-squares fitting procedure (XLfit, IDBS Ltd, Guildford, UK). The data were modelled from 2 minutes into the recovery process to allow for any muscle to sampling site time delay.

[BLa](t)=D+(A×exp(((−1)×(1/τ))×t)) Eqn 1

Where [BLa](t) is the lactate concentration at time t; D is the amplitude of the fitted model; A is the initial value of [BLa] minus D; and τ is the time constant of the response.

Venous blood samples were centrifuged at 3000 rpm for 10 minutes, with subsequently decanted plasma samples frozen at -80°C until analysed for caffeine content using high-performance liquid chromatography (HPLC). Before analysis, plasma samples were thawed, transferred to a separating flask, and made up to 2 mL with HPLC grade water. Following the addition of an internal standard, samples underwent solvent extraction using a chloroform/IPA mix (85%/15%). Each sample was extracted twice and the organic phase was removed each time. The organic phase from both extracts were subsequently combined, evaporated to dryness under nitrogen, and re-suspended in HPLC grade water. Analysis of caffeine content was carried out by reverse phase HPLC using a C18 column (Zorbax Eclipse Plus, Agilent Technologies Ltd., Stockport, UK) with a mobile phase of 80% water/20% methanol, a flow rate of 1.5 mL∙min^-1^, and ultra-violet detection at 274 nm.

### Statistical Analysis

All statistical analyses were conducted using the Statistical Package for the Social Sciences (SPSS for Windows; IBM Corporation, Armonk, New York, USA). Measures of centrality and spread are presented as means ± standard deviation. Differences between plasma samples in caffeine content were evaluated using a one-way repeated measures analysis of variance (ANOVA). The effects of caffeine supplementation on resting measures of [BLa] were evaluated using a two-way (supplement × time) repeated measures ANOVA; with effects on the other physiological measures, at rest and during the last 30 s of recovery, determined using paired-samples *t*-tests. The effects of supplementation on the time constants of the exponential models used to describe the recovery kinetics of [BLa] were also determined using a paired-samples *t*-test. Goodness of fit of the exponential models was evaluated using *F* tests. The effects of caffeine on physiological responses during the incremental tests, including the effects on [BLa] during recovery, were evaluated using two-way (supplement × exercise intensity or time) ANOVAs with repeated measures on both factors. α was set at 0.05 for all analyses. Violations to assumptions of sphericity were adjusted using the Greenhouse-Geisser correction factor. Significant interactions were followed-up using *post hoc* tests with Bonferroni adjustments for multiple comparisons. The above analyses provided 95% confidence limits for all estimates.

## Results

### Caffeine Consumption

Mean habitual caffeine consumption of the participants was estimated as 225 ± 135 mg∙d^-1^. Subject compliance with caffeine restriction prior to each trial was confirmed by the fact that in all non-caffeine supplemented conditions, plasma caffeine concentrations were low (0.25 ± 0.28 μg·mL^-1^), whereas values were high (3.90 ± 2.23 μg·mL^-1^) following caffeine.

### Blood Lactate

**Fig 1:**
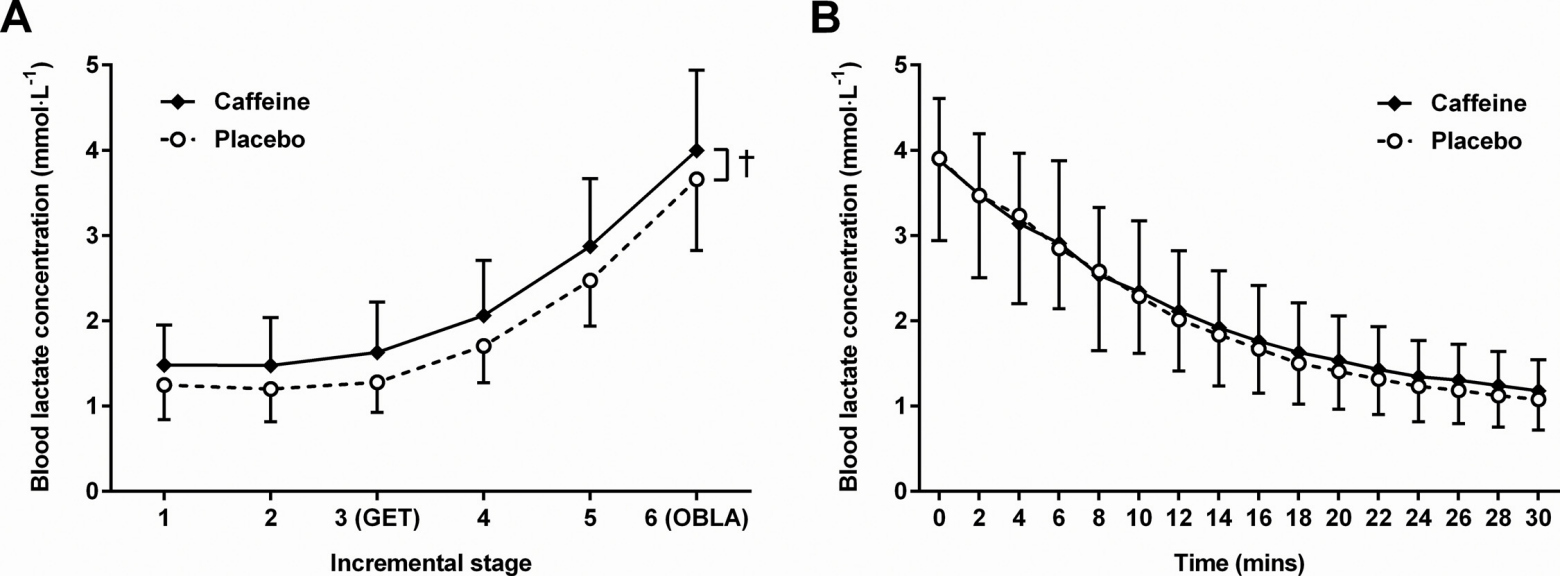
**The effects of caffeine supplementation on blood lactate concentration during submaximal incremental exercise (A); and in recovery from submaximal incremental exercise designed to achieve an end-exercise blood lactate concentration of 4 mmol∙L**^**-1**^ **(B). Values are means; bars are standard deviations**. GET = Gas exchange threshold; OBLA = Onset of blood lactate accumulation. † = significant main effect of supplement.

Blood lactate concentration decreased significantly (*F*_(1,15)_ = 15.87; *p* < 0.001) (mean difference: 0.20 ± 0.19 mmol∙L^-1^; 95% likely range: 0.11 to 0.29 mmol∙L^-1^) from pre-supplementation values of 1.24 ± 0.37 and 1.12 ± 0.40 mmol∙L^-1^ for caffeine and placebo trials respectively, to corresponding post-supplementation (pre-exercise) values of 1.07 ± 0.41 and 0.90 ± 0.34 mmol∙L^-1^. However, there was no effect of supplementation (*F*_(1,15)_ = 1.30; *p* = 0.263) and no supplement × time interaction (*F*_(1,15)_ = 0.25; *p* = 0.624). The effects of caffeine on [BLa] during exercise and recovery are presented in Fig 1. Relative to placebo, caffeine led to a significant increase (*F*_(1,15)_ = 10.13; *p* = 0.006) in [BLa] throughout the incremental tests of 0.33 ± 0.41 mmol∙L^-1^ (95% likely range: 0.11 to 0.55 mmol∙L^-1^); and there was a significant effect of exercise intensity on [BLa] (*F*_(1.3,19.6)_ = 181.57; *p* < 0.001). However, there was no significant supplement × exercise intensity interaction (*F*_(1.6,23.9)_ = 0.72; *p* = 0.466). In contrast, [BLa] decreased significantly over time during recovery (*F*_(1.6,23.4)_ = 213.28; *p* < 0.001), but there was no effect of supplementation on the recovery process (*F*_(1,15)_ = 0.28; *p* = 0.604), and no supplement × time interaction (*F*_(2.6,39.1)_ = 1.74; *p* = 0.181). The recovery kinetics of [BLa] were very well described by the mono-exponential models (mean F-test scores: caffeine = 1.010 ± 0.008; placebo = 1.013 ± 0.006); however, time constants were not significantly different (*t*_(15)_ = 0.53; *p* = 0.604) between the caffeine (τ = 14.8 ± 6.7 minutes) and the placebo (τ = 14.3 ± 6.0 minutes) conditions.

### Heart Rate

**Fig 2:**
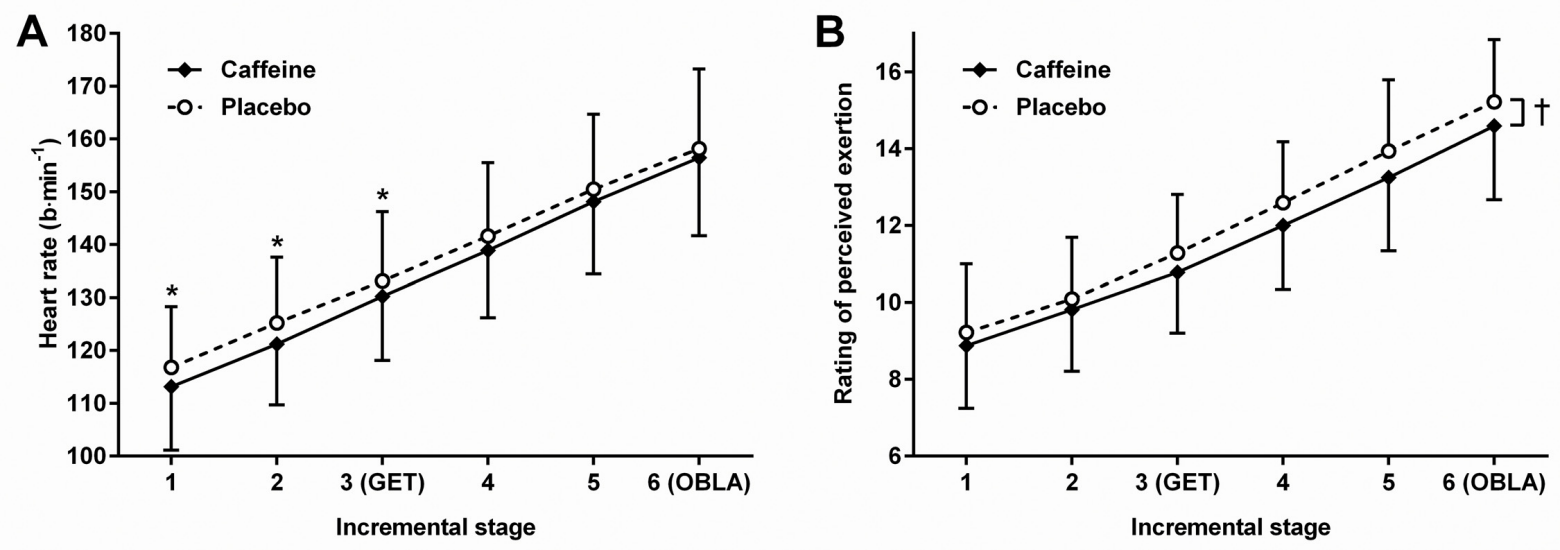
**The effects of caffeine supplementation on heart rate (A) and ratings of perceived exertion (B) during submaximal incremental exercise. Values are means; bars are standard deviations**. GET = Gas exchange threshold; OBLA = Onset of blood lactate accumulation. † = significant main effect of supplement; * = significant differences (*p* < 0.05) at the same exercise intensity.

**Fig 3:**
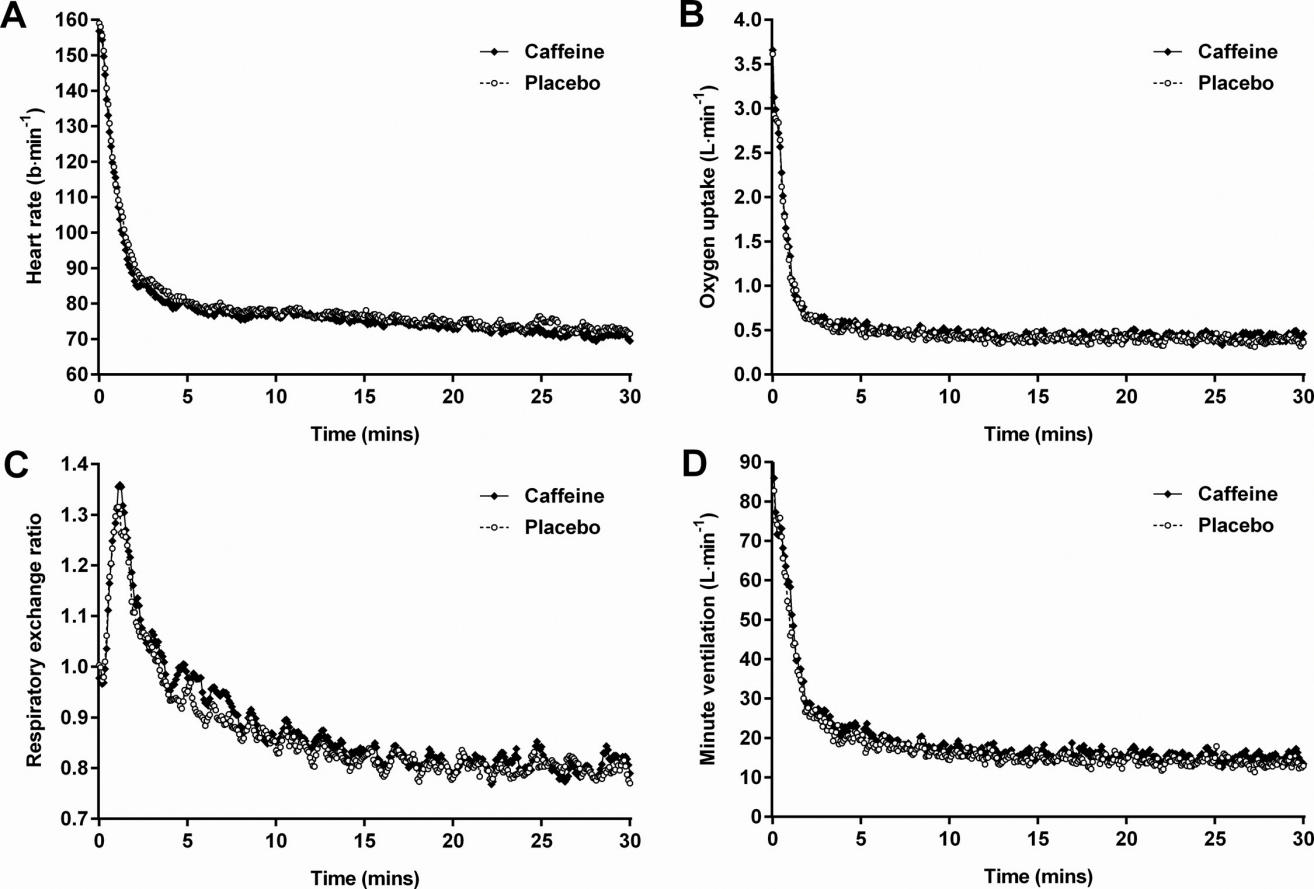
The effects of caffeine supplementation on heart rate (A), oxygen uptake (B), respiratory exchange ratio (C), and minute ventilation (D) measured at 5 s intervals during recovery from a bout of submaximal incremental exercise. Values are means.

The effects of caffeine supplementation on resting values of heart rate, $\dot{V}{\text{O}}_{2}$, RER, ${\dot{V}}_{\text{E}}$, and BF are presented in Table 1. Relative to placebo, caffeine supplementation led to a significant (*t*_(15)_ = 3.36; *p* = 0.004) reduction in heart rate at rest (mean difference: 3.6 ± 4.2 b∙min^-1^; 95% likely range: 1.3 to 5.8 b∙min^-1^) and during incremental exercise (*F*_(1,15)_ = 14.49; *p* = 0.002). However, the effect of caffeine on heart rate dissipated as exercise intensity increased (interaction effect: *F*_(5,75)_ = 2.49; *p* = 0.039). *Post hoc* tests revealed significant reductions in heart rate during the first three incremental stages only (Fig 2A). The effects of caffeine on the recovery of heart rate, $\dot{V}{\text{O}}_{2}$, RER, and ${\dot{V}}_{\text{E}}$ following the incremental tests in trials 2 and 3 are presented in Fig 3. After 30 minutes of recovery, heart rate was not significantly different (*t*_(15)_ = 0.88; *p* = 0.391) between caffeine and placebo conditions (Table 2).

**Table 1:** The effects of caffeine supplementation on resting measures of heart rate, oxygen uptake, respiratory exchange ratio, minute ventilation, and breathing frequency. Values are means ± standard deviations.

Supplement	Heart rate (b·min^-1^)	Oxygen uptake (L·min^-1^)	RER	Minute ventilation (L·min^-1^)	Breathing frequency (breaths·min^-1^)
Caffeine	59.5 ± 8.1*	0.45 ± 0.10*	0.84 ± 0.10*	14.21 ± 2.93*	14.4 ± 4.1
Placebo	63.0 ± 7.8	0.39 ± 0.08	0.81 ± 0.10	12.31 ± 3.55	14.6 ± 5.4

**Note**: RER = Respiratory exchange ratio.

* significantly different (*p* < 0.05) from placebo.

**Table 2:** The effects of caffeine supplementation on measures of blood lactate, heart rate, oxygen uptake, respiratory exchange ratio, and minute ventilation in the final 30 s of a 30 minute recovery period following submaximal incremental exercise. Values are means ± standard deviations.

Supplement	Blood lactate (mmol·L^-1^)	Heart rate (b·min^-1^)	Oxygen uptake (L·min^-1^)	RER	Minute ventilation (L·min^-1^)
Caffeine	1.38 ± 0.45	70.8 ± 9.0	0.43 ± 0.07*	0.82 ± 0.05	15.16 ± 2.21*
Placebo	1.27 ± 0.41	72.2 ± 10.5	0.36 ± 0.08	0.80 ± 0.04	13.35 ± 2.62

**Note**: RER = Respiratory exchange ratio.

* significantly different (*p* < 0.05) from placebo.

### Ratings of Perceived Exertion

Perceived exertion increased progressively during the incremental tests (*F*_(1.3,20.1)_ = 167.84; *p* < 0.001), and RPE was significantly lower (*F*_(1,15)_ = 8.81; *p* = 0.010) following caffeine supplementation than following placebo (mean difference: 0.5 ± 0.7; 95% likely range: 0.1 to 0.9). However, there was no supplement × exercise intensity interaction (*F*_(2.1,32.1)_ = 0.77; *p* = 0.48) (Fig 2B).

### Oxygen Uptake

**Fig 4:**
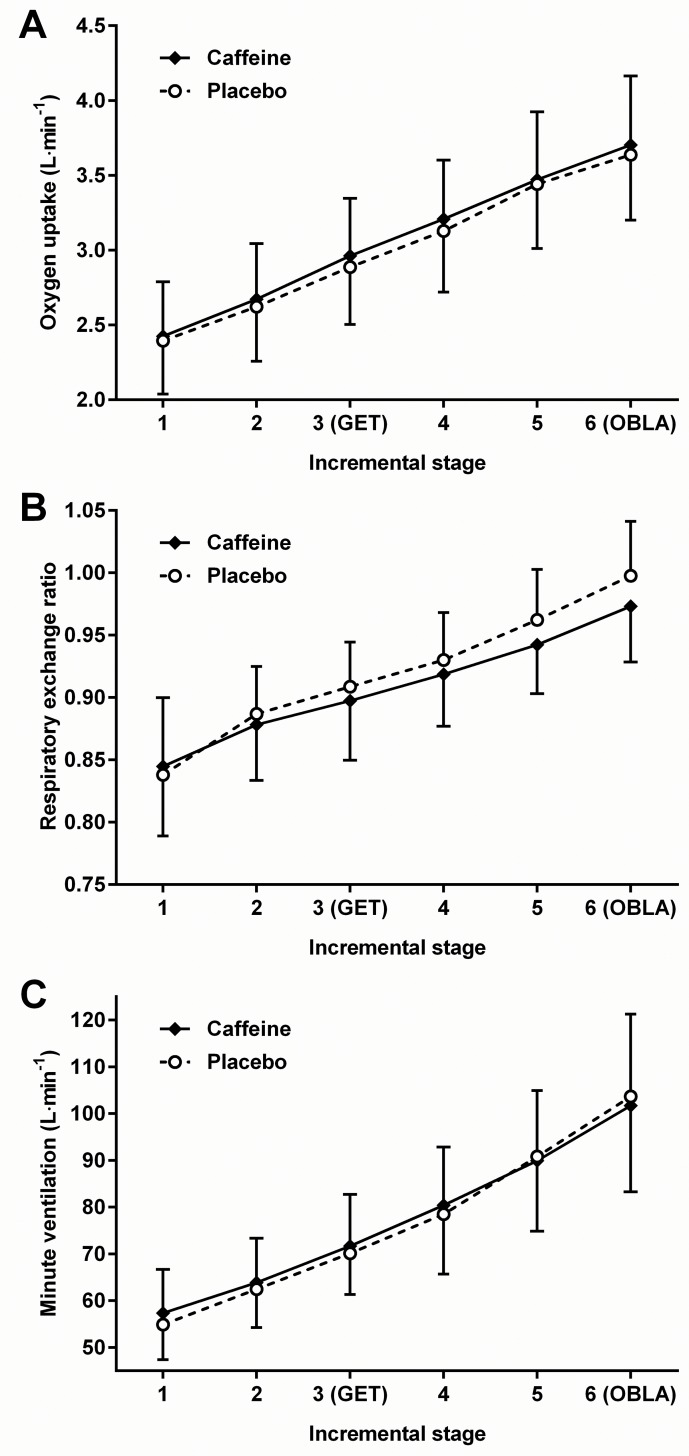
**The effects of caffeine supplementation on oxygen uptake (A), respiratory exchange ratio (B), and minute ventilation (C) during submaximal incremental exercise. Values are means; bars are standard deviations**. GET = Gas exchange threshold; OBLA = Onset of blood lactate accumulation.

There was a significant effect of exercise intensity on $\dot{V}{\text{O}}_{2}$ (*F*_(1.5,21.9)_ = 305.54; *p* < 0.001) and, in comparison with placebo, caffeine supplementation significantly increased (*t*_(15)_ = 2.51; *p* = 0.024) $\dot{V}{\text{O}}_{2}$ at rest (mean difference: 0.06 ± 0.09 L·min^-1^; 95% likely range: 0.01 to 0.11 L·min^-1^; Table 1) and at the end of 30 minutes of post-exercise passive recovery (mean difference: 0.07 ± 0.07 L·min^-1^; 95% likely range: 0.03 to 0.11 L·min^-1^; Table 2). However, relative to placebo, there was no significant effect of caffeine on $\dot{V}{\text{O}}_{2}$ during the incremental tests (*F*_(1,15)_ = 3.49; *p* = 0.082) (mean difference: 0.06 ± 0.12 L·min^-1^; 95% likely range: -0.01 to 0.12 L·min^-1^), and no supplement × exercise intensity interaction (*F*_(5,75)_ = 0.879; *p* = 0.499) (Fig 4A).

### Respiratory Exchange Ratio

Respiratory exchange ratio increased significantly during the incremental tests (*F*_(2.1,31.0)_ = 161.98; *p* < 0.001) and, in comparison with placebo, caffeine significantly increased (*t*_(15)_ = 2.25; *p* = 0.040) RER at rest (mean difference: 0.04 ± 0.06; 95% likely range: 0.001 to 0.07; Table 1). However, whilst there was no main effect of supplement during the incremental test (*F*_(1,15)_ = 2.17; *p* = 0.162), there was a significant supplement × exercise intensity interaction (*F*_(2.9,43.4)_ = 5.24; *p* = 0.004) (Fig 4B). Nevertheless, *post hoc* tests were unable to identify where those differences were. Moreover, relative to placebo, there was no significant effect (*t*_(15)_ = 1.23; *p =* 0.240) of caffeine on RER at the end of the post-exercise recovery period (Table 2).

### Minute Ventilation

Minute ventilation increased progressively during the incremental tests (*F*_(1.2,18.5)_ = 186.30; *p* < 0.001), and, in comparison with placebo, there was a significant increase (*t*_(15)_ = 2.44; *p* = 0.027) in ${\dot{V}}_{\text{E}}$ at rest (mean difference: 1.9 ± 3.1 L∙min^-1^; 95% likely range: 0.2 to 3.6 L∙min^-1^; Table 1), though not during exercise (*F*_(1,15)_ = 0.39; *p* = 0.543), following caffeine supplementation. Although there was a significant supplement × exercise intensity interaction (*F*_(2.6,39.2)_ = 3.36; *p* = 0.034) (Fig 4C), *post hoc* tests were unable to identify where those differences were. Nevertheless, relative to placebo, caffeine significantly increased ${\dot{V}}_{\text{E}}$ (*t*_(15)_ = 2.93; *p* = 0.011) at the end of the post-exercise recovery period (mean difference: 1.8 ± 2.4 L∙min^-1^; 95% likely range: 0.5 to 3.1 L∙min^-1^; Table 2).

### Breathing Frequency

Breathing frequency increased progressively throughout the incremental tests (*F*_(1.9,28.5)_ = 65.53; *p* < 0.001) from starting values (grand means) of 23.9 ± 3.4 breaths∙min^-1^ to end-exercise values of 32.8 ± 5.3 breaths∙min^-1^; however, relative to placebo, there was no effect of caffeine supplementation on BF either at rest (mean difference: 0.2 ± 4.1 breaths∙min^-1^; 95% likely range: -2.0 to 2.3 breaths∙min^-1^; Table 1) or during the incremental tests (*F*_(1,15)_ = 0.79; *p* = 0.387). Similarly, there was no supplement × exercise intensity interaction (*F*_(5,75)_ = 1.67; *p* = 0.152).

## Discussion

The aims of the present study were to evaluate the effects of caffeine supplementation on physiological responses to submaximal exercise, with a particular focus on the effects on [BLa]. The key findings were that relative to placebo, caffeine significantly increased [BLa] during exercise, but, when matched for the same end-exercise concentration, did not affect the rate of clearance in recovery. Moreover, there were significant effects on heart rate, RPE, and various respiratory measures, adding support to potential multifactorial whole-body effects of caffeine.

The significant effects of caffeine on [BLa] during incremental exercise were independent of exercise intensity. Given the absence of any effect of caffeine on [BLa] at rest, it appears that some degree of mechanical stress is required to achieve the response. As such, it seems unlikely that the caffeine-induced elevations in [BLa] can be attributed to an increased contribution from muscles that are not recruited during the exercise. Moreover, the absence of any effect of caffeine on the rate of [BLa] clearance in recovery suggests that the elevation in [BLa] was most likely due to an increase in lactate efflux from the working muscles, and thereby supportive of a caffeine-stimulated increase in glycolysis. While this suggestion conflicts with the findings of Graham et al. [21], at this point it is worth noting that in the present study, the effects of caffeine on [BLa] during exercise, though significant, were relatively small. As such, it is possible that the techniques used by Graham et al. [21] may have lacked sufficient sensitivity to detect changes of the magnitude indicated by the present study. Then again, in a study which combined the effects of a number of small sample size investigations, Graham et al. [36] found no difference between caffeine and placebo treatments on post-exercise (70–85% $\dot{V}{\text{O}}_{2\max}$) muscle glycogen concentrations. Given the relatively short duration of the exercise bouts (10–15 minutes) and the typical variability (~ 10%) in single muscle biopsy glycogen measurements [37], the effects of caffeine on glycogen depletion and lactate release in active muscle is clearly an area worthy of further investigation. Nevertheless, small changes in [BLa] coupled with the use of relatively small sample sizes may help to explain why significant effects of caffeine on [BLa] during submaximal exercise have been observed in some investigations [5,9,21,22], but not in others [4,13–15,18–20,25,26]; particularly given the fact that apart from Tarnopolsky et al. [20] and Wiles et al. [15], all of the latter reported a higher mean [BLa] response with caffeine.

Although the results of the present study support a positive effect of caffeine on glycolysis in active muscle during exercise, the mechanisms to explain that response are unclear [36]. At present, if true, the response seems most likely to be due to a direct effect on skeletal muscle via an antagonism of A_1_ adenosine receptors [36], and leading to a corresponding increase in intracellular cyclic adenosine monophosphate. Regardless of the mechanism, the response conflicts with any notion that caffeine has a glycogen-sparing mode of action. Then again, the idea of a caffeine-induced increase in glycolysis is difficult to reconcile with the absence, as in many previous studies [4,5,7,13,14,18,20–23,25], of a corresponding reduction in $\dot{V}{\text{O}}_{2}$. Indeed, caffeine led to a significant increase in $\dot{V}{\text{O}}_{2}$ at rest and at the end of recovery. In effect, these findings suggest that either caffeine is increasing energy metabolism irrespective of energy demand, or that caffeine is somehow causing a reduction in exercise efficiency. Given the established benefits of caffeine on aerobic-endurance based activities [1,2,3], the latter seems highly unlikely. In contrast, the idea of an increase in energy turnover forms part of the rationale for those studies that have investigated potential debilitative effects of caffeine on exercise tolerance in hot environments [38–42]. Despite a couple of exceptions [38,39], and a recurring theme of poor sample sizes and issues with experimental design [40]; there is little evidence of any caffeine-induced increases in metabolic rate or core temperature under those conditions [40–42]. Then again, and in contrast to the general consensus, those same studies have also mostly reported no effect of caffeine on sustained high-intensity aerobic exercise [38–40].

Although the effects of caffeine on [BLa] in the present study could be explained by a corresponding increase in glycolysis, it is difficult to reconcile that response with the pattern observed in RER. First, the significant caffeine-induced increase in resting measures of RER and $\dot{V}{\text{O}}_{2}$, despite no change in [BLa], suggests a disproportionate corresponding increase in $\dot{V}{\text{CO}}_{2}$. Secondly, the interaction between supplement and exercise intensity on RER suggests that the effect of caffeine on resting RER reversed as exercise intensity increased. Although *post hoc* tests were unable to locate significant differences resulting from the supplement × intensity interaction, a similar pattern of response in RER was observed by Stadheim et al. [13] across four fixed-intensity submaximal exercise bouts (40–70% $\dot{V}{\text{O}}_{2\max}$), with a significant effect being observed at the highest intensity. Moreover, although most previous research has failed to find an effect of caffeine on RER during single bouts of submaximal exercise [4,5,7,15,20,21,23,25], the trend, as in the present study, is towards a negative effect of caffeine on RER during exercise at moderate to high intensities [4,5,7,13,15,19–21,25]. Nevertheless, the result is at odds with the fact that increases in [BLa] are typically associated with increases in RER, particularly as submaximal exercise intensity increases [43]. Moreover, and in contrast to the effect at rest, the absence of a significant effect of caffeine on $\dot{V}{\text{O}}_{2}$ suggests that the effect on RER was due to a disproportionate reduction in $\dot{V}{\text{CO}}_{2}$ as exercise intensity increased.

It is difficult to resolve the mechanisms by which caffeine influenced the interaction effect on RER; however, it is possible that the effect was, at least in part, mediated by the corresponding supplementation × intensity interaction on ${\dot{V}}_{\text{E}}$. Previous research into the effects of caffeine on ${\dot{V}}_{\text{E}}$ during submaximal exercise has reported either a significant increase [4,7] or no effect [18–20]. In the present study, caffeine increased ${\dot{V}}_{\text{E}}$ at rest and, despite the absence of significant *post hoc* tests, the effect appeared to dissipate as exercise intensity increased. Moreover, the effect at rest returned in recovery. Given that effects were independent of any change in BF, changes in ${\dot{V}}_{\text{E}}$ appear to be largely the result of changes in tidal volume. The stimulatory effect of caffeine on ${\dot{V}}_{\text{E}}$ at rest has previously been observed in primates [44] and is attributed to a lowering of the sensitivity threshold for CO_2_ of central chemoreceptors; likely mediated by antagonism of adenosine receptors or, though less likely [17], by phosphodiesterase inhibition [44]. A caffeine-induced increase in ${\dot{V}}_{\text{E}}$ at rest would result in an increase in $\dot{V}{\text{CO}}_{2}$ and as such help to explain the corresponding increase in RER. Then again, it is difficult to reconcile that mechanism with the findings of McClaran & Wetter [45], who observed a significant reduction in RER at rest, albeit with a lower caffeine dose (3 mg∙kg^-1^), with no corresponding change in ${\dot{V}}_{\text{E}}$, or $\dot{V}{\text{O}}_{2}$. Moreover, in the present study, although the effect of caffeine on ${\dot{V}}_{\text{E}}$ showed signs of dissipating as exercise intensity increased (interaction effect), it is difficult to speculate as to how this response could explain the supplement × exercise intensity interaction on RER.

The caffeine-induced reduction in resting heart rate observed in the present study is of a magnitude similar to that previously reported [46]. Moreover, the response adds support to the idea that the effects of caffeine are mediated most likely by the antagonism of adenosine receptors, since adenosine infusion has been shown to increase resting heart rate by reducing parasympathetic and increasing sympathetic nervous system outflow [47]. Although the effect of caffeine on heart rate at rest did not return during recovery; given the pattern of the response (Fig 3A) and the fact that post-exercise heart rate did not return to pre-exercise resting values by the end of the 30 minute recovery period, it is possible that the effect would have returned if the recovery period had been extended. In contrast, the dissipation of the effect of caffeine on heart rate as exercise intensity increased was not anticipated, but provides an explanation as to why previous research, using continuous bouts of submaximal exercise at intensities similar to those at the upper end of the profile used in the present study, have failed to find an effect of caffeine on heart rate [4,5,7,9,11,13,14,18,20–23,25]. Indeed, the results of the present study suggest that the caffeine-induced elevations in heart rate that have been observed in some performance-based studies are likely to be due to the correspondingly higher exercise intensities maintained by the participants rather than from any direct stimulatory effect of caffeine [6,8,11,13,14].

Although the effects of caffeine on heart rate dissipated as exercise intensity increased, RPE was suppressed by caffeine throughout the incremental protocol. Given that the RPE scale was developed to reflect heart rate responses to exercise [29], the results of the present study suggest that caffeine may have some effect on the stability of that relationship. Nevertheless, the results of the present study confirm previous reports of a suppressive effect of caffeine on RPE during constant-load submaximal exercise [48]; an effect that is likely to be due to the antinociceptive effects of caffeine, mediated centrally by antagonism of A_1_ adenosine receptors [17]. Indeed, the antinociceptive effects of caffeine may explain many, if not all, of the subsequent performance enhancing effects [16,48].

## Conclusion

Despite many years of research, the effects of caffeine supplementation on physiological responses to exercise remain unresolved, most likely because of issues associated with small sample sizes and the associated risks of type 2 statistical errors. Moreover, the complex central and peripheral effects of caffeine, which appear to be associated predominantly with the antagonism of the various adenosine receptors subtypes, make it difficult to discern mechanisms of action. Nevertheless, the results of the present study support a caffeine-induced increase in [BLa] during exercise; an effect which appears to be independent of exercise intensity and which, though easily explained by a stimulation of glycolysis, clearly requires more research to clarify. In addition, caffeine reduced resting heart rate and increased resting $\dot{V}{\text{O}}_{2}$, RER, and ${\dot{V}}_{\text{E}}$. Although the effects of caffeine on heart rate, RER and ${\dot{V}}_{\text{E}}$, were influenced additionally and differentially by exercise intensity, it is difficult to discern if any of these effects are responsible for the ergogenic action of caffeine. Finally, caffeine reduced RPE during exercise, again independently of exercise intensity, and this effect remains one of the key factors which may explain the benefit of caffeine on sustained high-intensity aerobic exercise.
